# Hidden in the daylight: A polyphasic approach reveals two bioluminescent Mycenaceae species from Yunnan Province, China (Basidiomycota, Agaricales)

**DOI:** 10.3897/BDJ.13.e168858

**Published:** 2025-12-18

**Authors:** Wenhua Lu, Alanoud T. Alfagham, Saowaluck Tibpromma, Nakarin Suwannarach, Jaturong Kumla, Dong-Qin Dai, Abdallah M. Elgorban, Ekachai Chukeatirote, Samantha C. Karunarathna

**Affiliations:** 1 Excellence Center of Microbial Diversity and Sustainable Utilization, Chiang Mai University, Chiang Mai 50200, Thailand Excellence Center of Microbial Diversity and Sustainable Utilization, Chiang Mai University Chiang Mai 50200 Thailand; 2 Center for Yunnan Plateau Biological Resources Protection and Utilization & Yunnan International Joint Laboratory of Fungal Sustainable Utilization in South and Southeast Asia, College of Biology and Food Engineering, Qujing Normal University, Qujing 655099, China Center for Yunnan Plateau Biological Resources Protection and Utilization & Yunnan International Joint Laboratory of Fungal Sustainable Utilization in South and Southeast Asia, College of Biology and Food Engineering, Qujing Normal University Qujing 655099 China; 3 Department of Biology, Faculty of Science, Chiang Mai University, Chiang Mai 50200, Thailand Department of Biology, Faculty of Science, Chiang Mai University Chiang Mai 50200 Thailand; 4 Department of Botany and Microbiology, College of Science, King Saud University, Riyadh 11451, Saudi Arabia Department of Botany and Microbiology, College of Science, King Saud University Riyadh 11451 Saudi Arabia; 5 Office of Research Administration, Chiang Mai University, Chiang Mai 50200, Thailand Office of Research Administration, Chiang Mai University Chiang Mai 50200 Thailand; 6 Center of Excellence in Biotechnology Research (CEBR), King Saud University, Riyadh 11451, Saudi Arabia Center of Excellence in Biotechnology Research (CEBR), King Saud University Riyadh 11451 Saudi Arabia; 7 School of Science, Mae Fah Luang University, Chiang Rai 57100, Thailand School of Science, Mae Fah Luang University Chiang Rai 57100 Thailand

**Keywords:** bioluminescent fungi, diversity, ecology, *Mycena semivestipes*, *Roridomyces pruinosoviscidus*, saprobic fungi

## Abstract

**Background:**

Yunnan Province, recognised as a biodiversity hotspot in China, is a rich source of fungal diversity that we are just beginning to explore. During our investigation of bioluminescent mushrooms in this region, we collected four specimens from the family Mycenaceae. We conducted morphological characterisation, multilocus phylogenetic analyses (ITS, LSU, SSU, *tef*1-α and *rpb*2) and photographed specimens under completely dark conditions.

**New information:**

In this paper, we report on two bioluminescent mushrooms of Mycenaceae collected in Yunnan Province, China. Morphological and phylogenetic analyses revealed *Mycena semivestipes*, which was first observed with bioluminescence and *Roridomyces pruinosoviscidus*, which has not been previously recorded in China. Additionally, we provide descriptions, illustrations, phylogenetic analysis results and photographs that highlight the bioluminescent features of these taxa. This study enhances our understanding of bioluminescent mushroom diversity in the Yunnan Province, raising the total number of bioluminescent mushrooms reported from China to 36.

## Introduction

Yunnan Province, situated in southwest China, is one of the regions with the richest biodiversity in the country. Its unique geographical environment and climatic conditions support a rich and diverse array of biological resources (Zhang et al. 2021). However, despite the increasing research on plant and animal diversity, the understanding of fungal diversity remains understudied (Feng and Yang 2018, Lu et al. 2024a). Bioluminescent fungi, a remarkable group of organisms within the fungal kingdom, have garnered significant attention for their potential applications in ecology, medicine and genetic engineering (Syed and Anderson 2021, Lu et al. 2024b). Bioluminescence is not uncommon, but its occurrence in fungi is relatively rare. Fungal bioluminescence is produced by a special biochemical reaction, which usually involves luciferin, luciferase and oxidation (Kotlobay et al. 2018, Lu et al. 2024b). Kotlobay et al. (2018) reported that the fungal bioluminescence biosynthesis pathway involves fungal luciferase and three other key enzymes in a cycle of caffeic acid. The new view emerged by integrating gene editing technology with plant cultivation, enabling the transfer of mushroom genes into living organisms (Mitiouchkina et al. 2020, Zheng et al. 2023, Ge et al. 2024). However, the role of mushroom bioluminescence in ecology remains a mystery.

First, some researchers believe that fungi emit light to attract phototactic insects, thereby assisting in spore dispersal, especially in deep forest areas where wind is blocked (Lu et al. 2024b). Creating artificial mushrooms with light has been shown to attract more insects than non-luminous mushroom models (Oliveira et al. 2015). Weinstein et al. (2016) scrutinised the abundance of insects recorded in the field during basidiomata production and they evaluated the allure of bioluminescent fungi to flying insects, finding no discernible difference in *Omphalotus nidiformis* (Berk.) O.K. Mill. Second, the bioluminescence might arise as an incidental by-product of metabolism rather than providing any distinct evolutionary advantage (Nimalrathna et al. 2022, Lu et al. 2024b). The bioluminescent parts vary significantly amongst different species; *Armillaria* (Fr.) Staude species exhibit bioluminescence during the mycelium stage, while in other species, bioluminescence occurs on the pileus, gills, stipe or spores (Lu et al. 2024b). During the development of *Armillaria* from mycelium to mushroom, it has been observed that the synthesis of luciferin precursors and hispidin-3-hydroxylase was inhibited (Mihail et al. 2018). Additionally, some studies hypothesise whether bioluminescent mushrooms generate oxidative stress responses to counteract the oxygen released during the respiratory phase, as the bioluminescent mechanism also entails oxygen consumption (Briones-Martin-Del-Campo et al. 2014, Warris and Ballou 2019, Yaakoub et al. 2022, Emri et al. 2024).

Moreover, all bioluminescent mushrooms are found in tropical, subtropical and temperate regions (Lu et al. 2024b). Mushrooms dissipate excess absorbed energy to maintain internal energy balance. Bioluminescence — often referred to as 'cold light' (Cordero et al. 2023) — may serve this function, suggesting it is an evolutionary adaptation for energy regulation. This phenomenon ensures that mushrooms do not overheat due to absorbed energy, which could potentially harm their cellular structures or disrupt metabolic processes (Iqbal et al. 2009, Tisch and Schmoll 2010). Instead, they convert this excess energy into visible light, a process crucial for survival in their respective habitats. However, further investigation is necessary to elucidate the significance of the mushroom bioluminescence. There are more than 132 fungal species that exhibit bioluminescence properties, all of which belong to the Basidiomycota, except for *Xylaria hypoxylon* (L.) Grev, which belongs to the phylum Ascomycota and, amongst them, the family Mycenaceae Overeem contains most bioluminescent mushrooms worldwide (Lu et al. 2024b, Perry et al. 2025).

*Mycena* (Pers.) Roussel is the largest genus in Mycenaceae, comprising more than 500 species distributed worldwide (Nagamune et al. 2024, Soares et al. 2024). It plays a key role in the circulation of forest ecosystems, as saprobic fungi dominate the degradation of fibres synergistically with endophytic fungi and some species contain biologically active and antimicrobial compounds (Bäuerle et al. 1982, Frankland 1998, Fukasawa et al. 2009). The genus *Mycena* is characterised by small, multicoloured basidiomata; pileus conical, parabolic and bell-shaped, hygrophanous, membranous with or without striations on the surface and smooth; lamellae adnate, decurrent or arcuate; stipe cylindrical, hollow and fragile; cystidia with protuberances, various shapes, clavate, utriform, pyriform, fusiform, hyaline, amyloid and thin-walled, containing oil; and spores globose, subglobose, ellipsoid, cylindrical and occasionally ovoid (Seok et al. 2015, Qiang and Bai 2023, Nagamune et al. 2024, Wei et al. 2024). This genus comprises more than 80 described or reported bioluminescent mushroom species, the largest number of bioluminescent species known worldwide (Chew et al. 2014, Heinzelmann et al. 2024, Lu et al. 2024b, Soares et al. 2024, Perry et al. 2025).

*Roridomyces* Rexer was established by Rexer (Rexer 1994) with the type species *R. roridus* (Fr.) Rexer, characterised by pileus trama, composed of interwoven, cylindrical hyphae and the pileipellis, a hymeniderm composed of clavate to subglobose terminal elements. There are 15 species worldwide; amongst them, six are bioluminescent mushrooms. Seven *Roridomyces* species have been reported from China, viz. *R. appendiculatus* Rexer, *R. glutinosus* (Corner) T. Bau & L.N. Liu, *R. mauritianus* (Robich & Hauskn.) Hauskn. & Krisai, *R. praeclarus* (E. Horak) Rexer, *R. lamprosporus* (Corner) Rexer, *R. roridus* and *R. viridiluminus* L.A.P. Dauner, Karunarathna & P.E. Mortimer (Dauner et al. 2021), of which the last three species are bioluminescent (Dauner et al. 2021, Lu et al. 2024b).

As mentioned above, the peculiar biological phenomenon of bioluminescence is of great significance in biological research and has attracted widespread interest. For this reason, understanding the diversity of bioluminescent fungi and their ecological characteristics in Yunnan is crucial, but remains limited to date (Lu et al. 2024b). This study reports two bioluminescent mushrooms in the Mycenaceae, based on a polyphasic approach: *Mycena semivestipes* (Peck) A.H. Sm., reported here for the first time for its bioluminescence and *Roridomyces pruinosoviscidus* (Corner) Blanco-Dios, a new geographical record for China. These findings contribute to our understanding of bioluminescent mushroom diversity from China, increasing the total number of known bioluminescent mushroom species in the region to 36. Additionally, the biological significance of bioluminescence is discussed.

## Materials and methods

### Sample collection, pure culture isolation and herbarium specimen preparation

Fresh basidiomata that morphologically resemble Mycenaceae were collected from detached pieces of dead and rotting wood near a mountain stream in a wet forest in Yunnan Province, southwest China, in July 2023. All the important field information (altitude, colour, date, GPS coordinates, habitat and substrate) associated with the mushrooms was noted (Rathnayaka et al. 2024). The specimens were photographed *in situ* in daylight and in the laboratory using a Huawei P50 Pro camera (Shenzhen, P.R. China). The bioluminescent photographs were taken at night with a Canon EOS 80D camera (Tokyo, Japan) set to f/5.6, ISO 3200 and a shutter speed of 90 seconds in a black box. The basidiomata were then returned to the mycology laboratory at Qujing Normal University, where the specimens were completely dried in a hot air oven at 40℃ (Hu et al. 2022). The pure cultures were obtained using the spore print technique; the fresh mushroom caps were stuck on a sterile cover Petri plate with potato dextrose agar (PDA) and, after ten minutes, the mushroom caps with the Petri plate cover were removed; a new Petri plate cover was replaced and incubated for 24 h at 28℃. Germinated spores were transferred to a new PDA plate and incubated in an incubator (28℃) to observe and photograph bioluminescence and to facilitate further DNA extraction. All dried specimens were deposited in the Guizhou Medical University Herbarium (GMB) and living cultures were deposited in the Guizhou Medical University Culture Collection (GMBCC).

### Morphological study

Macromorphological characteristics were described using the terminology of Largent (1986), based on notes from fresh collections and associated photographs. Kornerup and Wanscher’s colour terms and code were followed in this study (Kornerup and Wanscher 1967). The microscopic characteristics were described by using the concepts of Largent et al. (1977). Micromorphological observations were conducted using the methods described by Lu et al. (2024a). Freehand sections of the dried specimens were mounted in 5% potassium hydroxide (KOH) and stained with Congo red for microscopic examination. Melzer’s reagent was used to enhance contrast and assess the amyloid reaction in basidiospores. The light Eclipse 80i microscope (Olympus, Japan) was used to examine various features, including basidia, basidiospores, cystidia and hyphae. Measurements were conducted on at least 50 spores and their sizes were determined. Basidiospore dimensions were denoted as (a–) b–c (–d), where the range ‘b–c’ encompassed 90% or more of the measured values, with a and d representing the extreme values. Parameter Q refers to the interval of the length/width ratio of all basidiospores measured. Qm represented the mean Q value with standard deviation. Detailed illustrations of microstructures were sketched by hand using rehydrated materials and subsequently refined using Adobe Illustrator 2019.

### DNA extraction, PCR amplification and sequencing

The total DNA was extracted from dried specimens and pure cultures using the Biospin Fungus Genomic DNA Extraction Kit-BSC14S1 (BioFlux, P.R. China), according to the manufacturer’s instructions, with minor modifications. The internal transcribed spacer region (ITS), large subunit (LSU) and small subunit (SSU) of the cistron coding for the ribosomal rRNAs and two protein-coding genes, translation elongation factor 1-α (*tef*1-α) and RNA polymerase II (*rpb*2), were amplified using the primer pairs ITS1/ITS4, LR0R/LR5, NS1/NS4, 983F/2218R and 5F/7cR, respectively (Vilgalys and Hester 1990, White et al. 1990, Liu et al. 1999), in a total reaction volume of 25 μl containing 12.5 μl of 2x Master Mix (mixture of Easy Taq TM DNA Polymerase, dNTPs and optimised buffer (Beijing Trans Gen Biotech Co., Chaoyang District, Beijing, P.R. China), 8.5 μl distilled water, 2 μl DNA template and 1 μl of each primer. The cycle parameters were as follows: an initial denaturation at 94℃ for 5 min; denaturation at 94℃ for 30 s, annealing at 54℃ for 40 s (ITS, LSU, SSU and *tef*1-α) or at 58℃ for 90 s (*rpb*2) and an extension at 72℃ for 1 min for 35 cycles; with a final extension at 72℃ for 10 min; storage at 4℃. PCR products were sent to Sangon Biotech Co., Ltd. (Kunming, China) for sequencing with the PCR primers mentioned above. All newly-generated sequences in the present study were deposited in GenBank (https://www.ncbi.nlm.nih.gov/genbank, assessed on 25 September 2024).

### Phylogenetic analyses

Raw sequence reads (forward and reverse) were checked and manually edited for quality in BioEdit version 7.0.5 (Hall 1999) to correct base-calling errors, trim low-quality ends and confirm sequence orientation. Contigs were then assembled using Sequencher version 5.4.6 (Gene Codes Corporation 2016). The most similar sequences were identified using BLASTn (https://blast.ncbi.nlm.nih.gov/Blast.cgi, accessed on 10 June 2025) and were downloaded for phylogenetic analyses. Each dataset (ITS, LSU, SSU, *tef*1-α and *rpb*2) was aligned using MAFFT version 7 on the online server platform (www.ebi.ac.uk/Tools/mafft; Katoh and Standley (2013)). The “auto” strategy was employed, allowing MAFFT to select the optimal alignment algorithm for each dataset. TrimAL version 1.2 (http://trimal.cgenomics.org, accessed 10 June 2025) was used to automatically remove gaps and ambiguous regions, applying the gt 0.4 algorithm. The final alignments for each locus were concatenated using SequenceMatrix v.1.7.8 to generate the multi-gene dataset. The final FASTA format was converted to PHYLIP and NEXUS formats using the Alignment Transformation Environment (ALTER) online programme (Glez-Peña et al. 2010). Prior to multi-locus analyses, single-gene tree topologies were inspected for topological conflicts at nodes with bootstrap support > 70%; none was observed. A concatenated dataset of ITS, LSU, SSU, *tef*1-α and *rpb*2 was assembled in BioEdit v.7.0.5, with gene regions concatenated in the order listed and partitioned by gene region, resulting in five character sets. Partitioned Maximum Likelihood (ML) analyses were conducted in RAxML-HPC2 v.8.2.12 (Stamatakis 2014) via the CIPRES Science Gateway v.3.3 (http://www.phylo.org/portal2, accessed on 13 November 2025) (Miller et al. 2010). A partitioned (mixed) model was implemented in RAxML using a partition file, with each gene region assigned its own GTRGAMMA substitution model. Node support was assessed with 1,000 rapid bootstrap replicates.

Bayesian Inference (BI) analyses were conducted in MrBayes version 3.2.7a via the same web portal as the ML analyses (Ronquist et al. 2012). The analyses ran on XSEDE computational resources. Two independent runs of six Markov chains each for 20,000,000 generations, sampling every 200 generations (100,000 samples per chain) were undertaken. Preliminary inspection of an initial exploratory MCMC anlysis indicated that stationarity was not reached within the 2,000,000–5,000,000 generations; the final MCMC analysis was, therefore, run for 20,000,000 generations to achieve adequate mixing and convergence. Convergence and stationarity were assessed from both independent runs using three criteria: (1) the average standard deviation of split frequencies (ASDSF), which reached 0.009991 (threshold < 0.01); (2) effective sample sizes (ESS) of combined post-burn-in parameter traces, all of which exceeded 200, with most parameters showing ESS values well above 1,000, as confirmed in Tracer v.1.7.2 (Rambaut et al. 2018); and (3) log-likelihood and parameter trace plots exhibited stable, stationary “caterpillar”-like patterns with no observable trends and the two independent runs showed highly overlapping traces. Substitution models for each partition were selected under the Akaike Information Criterion (AIC) using MrModelTest v.2.2 (Nylander 2004). In these final runs, burn-in was assessed by visually inspecting the log-likelihood and parameter traces and all pre-stationary samples were removed. Examination of the Tracer plots indicated that stationarity was reached at ~ 1.0–1.2 million generations and we, therefore, conservatively discarded the first 10% of samples as burn-in. A 50% majority-rule consensus tree was constructed from the post–burn-in trees, retaining clades present in ≥ 50% of samples, with posterior probabilities provided for each clade. Phylogenetic trees were visualised using FigTree version 1.4.0 (Rambaut 2009) and were edited in Microsoft PowerPoint. The reliable bootstrap support values of ML (BS ≥ 70%) and Bayesian posterior probabilities (PP ≥ 0.90) were indicated above each branch.

## Taxon treatments

### Mycena semivestipes

(Peck) A.H. Sm., 1947

17A8A0AE-913C-5E79-A5FD-FA3BFC93F49A

288544

#### Materials

- **Type status:** Other material. **Occurrence:** occurrenceID: 8DC70ECC-4DD5-5DDE-BE1A-B9A03672DE9E; **Taxon:** kingdom: Fungi; phylum: Basidiomycota; class: Agaricomycetes; order: Agaricales; family: Mycenaceae; genus: Mycena; taxonRank: species; **Location:** country: China; stateProvince: Yunnan; county: Chuxiong; verbatimElevation: 1871 m; verbatimLatitude: 24°54′31″ N; verbatimLongitude: 101°07′25″ E; **Identification:** identifiedBy: Wenhua Lu; **Event:** year: 2023; month: July; day: 15; **Record Level:** institutionID: Guizhou Medical University Herbarium (GMB); institutionCode: GMB1045; collectionCode: CX006; basisOfRecord: other material GMB1047, living culture GMBCC1066; GenBank accession numbers ITS: PQ373879 (GMB1045), PQ373880 (GMB1047), PQ373883 (GMBCC1066); LSU: PQ373885 (GMB1045), PQ373886 (GMB1047), PQ373889 (GMBCC1066)

#### Description

**Figure 1.**
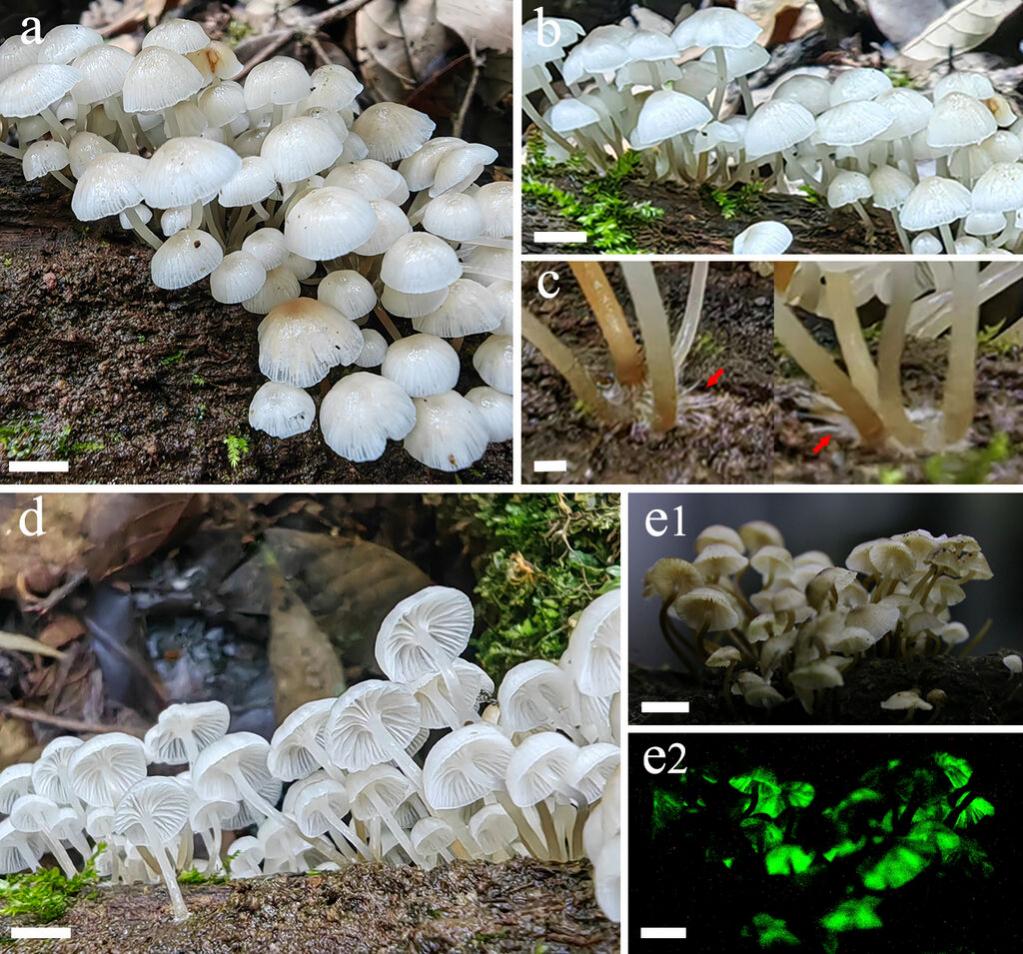
Basidiomata of *Mycena semivestipes* (GMB1045). **a, b, d** Photographs in the daylight; **c** Stipe with white mycelium hairs (red arrow); **e1** Photograph with the aid of a flashlight in the lab; **e2** Bioluminescent photographs in complete darkness. Scale bars: a, b, d, e = 10 mm, c = 1 mm.

**Figure 2.**
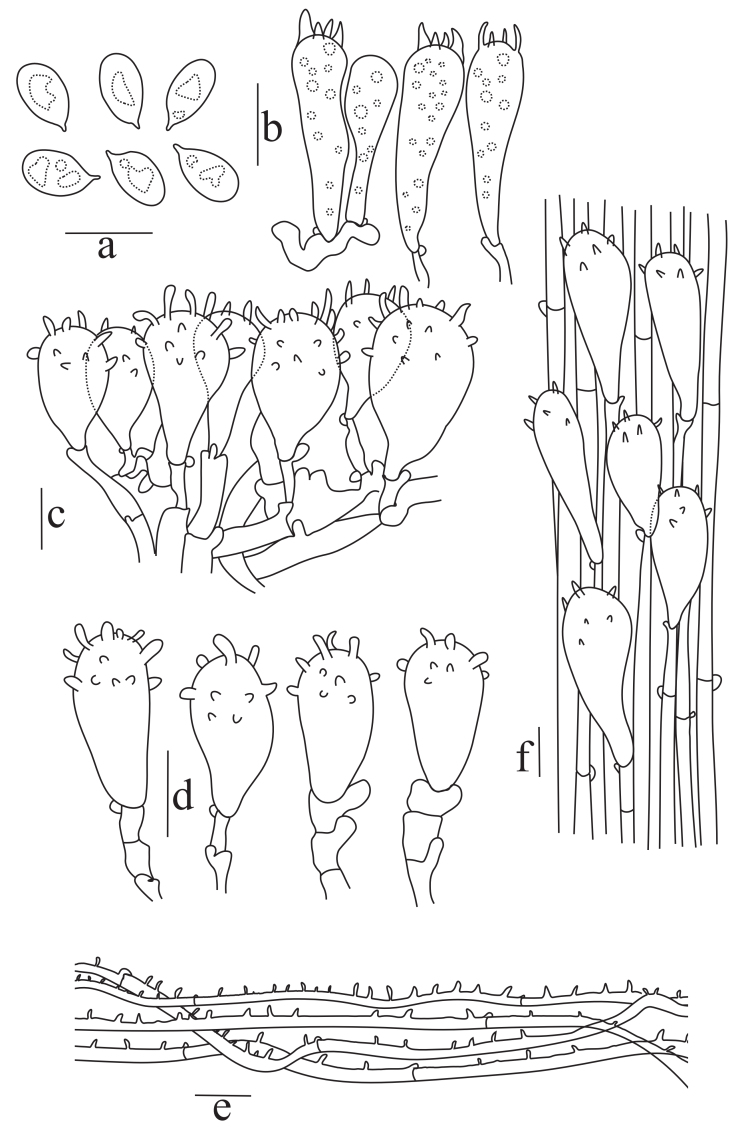
Microscopic characteristics of *Mycena semivestipes* (GMB1045). **a** Spores; **b** Basidia; **c** Cheilocystidia; **d** Pleurocystidia; **e** Pileipellis hyphae; **f** Stipitipellis with caulocystidia. Scale bars = 10 µm.

**Basidiomata** small. **Pileus** 3–15 mm diam., hemispheric, campaniform, expanding to flatten with age, initially convex to obtusely conical, margin white, centre greyish-white (1B1) when young, surface wet, smooth, slimy, depressed, striate-plicate, translucid; centre light orange (5A4–5); margin white or translucid, thin, translucent-striate, hygrophanous in age; colour changes to greyish-orange (5B4–5), brownish-orange (5C5–6) when mature. **Context** thin, fragile, translucent, white. **Lamellae** adnate to subdecurrent, whitish to white with 1–3 series of lamellulae, edges finely fimbriate under a lens, concolorous. **Stipe** 20–45 × 0.6–1.2 mm, cylindrical, hollow, brittle, brownish-orange to reddish-golden (6C7–8), milk-white (1A1–2) from base upwards, brown (6E7–8) at the base with whitish mycelium fascicles. **Smell and taste** none (Figs 1, 2).

**Figure 3.**
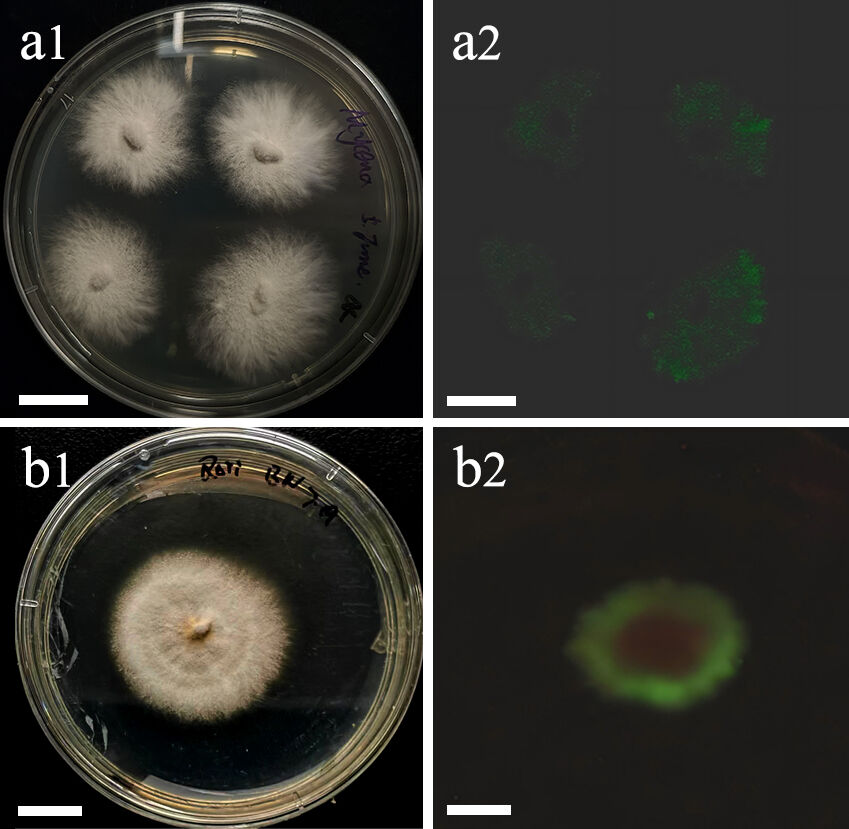
Cultures of bioluminescent fungi on PDA in daylight (a1 and b1) and dark conditions (a2 and b2) after seven days incubation at 25–28 ℃. **a** *Mycena semivestipes* (GMBCC1066); **b** *Roridomyces pruinosoviscidus* (GMBCC1068). Scale bars = 10 mm.

**Bioluminescence**: Only the caps and gills of basidiomata and the mycelia on the PDA emit yellowish-green light and the bioluminescence on spores was undetected (Fig. 3a).

**Basidiospores** (3.4–) 4.0–5.0 (–5.5) × (2.0–) 2.3–2.8 (–3.0) μm (n = 50), Q = 1.55−1.80, Qm = 1.7, ellipsoid to oblong, smooth, hyaline, containing oil droplets, thin-walled, inamyloid. **Basidia** 14.5–22 × 4–6.5 μm (n = 15), 4-sterigmata, clavate, some with oily contents, thin-walled. **Cheilocystidia** abundant, 15–35 × 7.5–13 μm, mainly clavate, cylindrical to fusiform, utriform with warty or finger-like protuberances, colourless, thin-walled. **Pleurocystidia** like cheilocystidia, 14–33.5 × 8–13 μm. **Pileipellis** a cutis with a well-developed cutis structure, hyphae 4–6 μm wide, cylindrical, parallel, with abundant, numerous sharp spines, colourless, thin-walled. **Stipitipellis** is a cutis with thin-walled hyphae. **Caulocystidia** clavate or fusiform. **Clamp connections** present on all hyphae and structures are abundant (Figs 1, 2).

#### Distribution

Canada, China, France, Sweden and the USA.

#### Ecology

Scattered, clustered or in groups on decaying trees in mixed coniferous and broad-leaved forests in summer and autumn.

#### Notes

The ITS sequences of our *Mycena* collections had the highest BLAST score to that of *M. semivestipes* (LE-BIN 3362) with 98.62% (difference in three indels and three substitutions) and *M. tintinnabulum* (NSK 1017255) with 99% (difference in three indels and four substitutions). Morphologically, both *M. semivestipes* and *M. tintinnabulum* are very similar; the former differs in that the finger-like protuberances of cystidia are sparse, whereas in *M. tintinnabulum*, cheilocystidia bear numerous short finger-like or occasionally branched projections, giving a distinctly ornamented appearance (Smith 1937, Smith 1947, Na 2019). *Mycena tintinnabulum* is mainly found in Europe and the bioluminescence is present only in the mycelium. Our collections are consistent with *M. semivestipes* in many cases (small, lubricous basidiomata, adnate to subdecurrent lamellae, smooth amyloid spores, gelatinous pileipellis, cartilaginous context and cheilocystidia smooth or somewhat contorted and branched, but not numerous finger-like processes); Na (2019) reported that it is a new record for China, based on specimens from southwest China. Macro-morphological characteristics indicate that the colour of the basidiomata in our collection is similar to that of the Chinese specimen *M. semivestipes*, which is white or dirty white and changes to a yellowish-brown to light brown after being removed from the habitat. However, the fruiting bodies of European species are dark brown or nearly dark brown.

### Roridomyces pruinosoviscidus

(Corner) Blanco-Dios, 2020

05F0B69F-2E7A-5B9B-8E10-F475152D265E

557509

#### Materials

- **Type status:** Other material. **Occurrence:** occurrenceID: 62EA86DA-A16C-5000-B38F-009ECD731B09; **Taxon:** kingdom: Fungi; phylum: Basidiomycota; class: Agaricomycetes; order: Agaricales; family: Mycenaceae; genus: Roridomyces; specificEpithet: pruinosoviscidus; taxonRank: species; **Location:** country: China; stateProvince: Yunnan; county: Xishuangbanna; locality: Xishuangbanna Primitive Forest Park; verbatimElevation: 1280 m; verbatimLatitude: 21°59′32″ N; verbatimLongitude: 100°54′44″ E; **Identification:** identifiedBy: Wenhua Lu; **Event:** year: 2023; month: July; day: 7; **Record Level:** institutionID: Guizhou Medical University Herbarium (GMB); institutionCode: GMB1054; collectionCode: LWH2307; informationWithheld: other material, GMB1056, living culture GMBCC1068; GenBank accession numbers ITS: PQ373881 (GMB1054), PQ373882 (GMB1056), PQ373884 (GMBCC1068); LSU: PQ373887 (GMB1054), PQ373888 (GMB1056), PQ373890 (GMBCC1068)

#### Description

**Figure 4.**
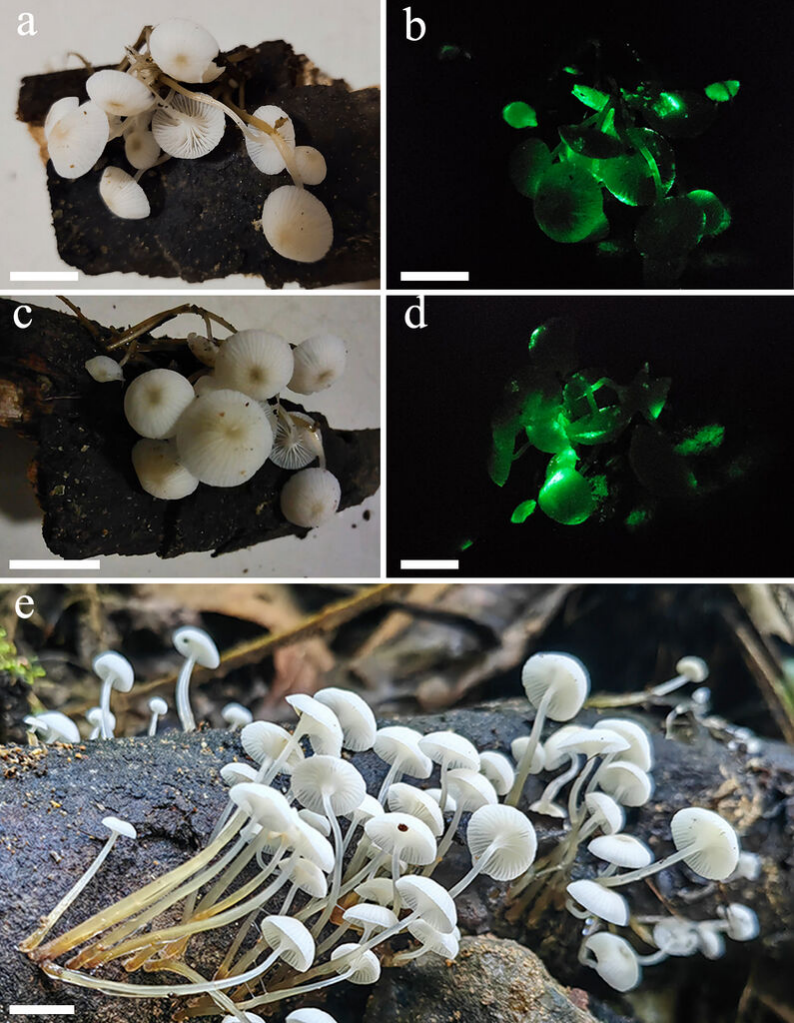
Basidiomata of *Roridomyces pruinosoviscidus* (GMB1054). **a, c, e** Photographs in daylight in a room and the field; **b, d** Bioluminescent photographs in complete darkness. Scale bars: 10 mm.

**Figure 5.**
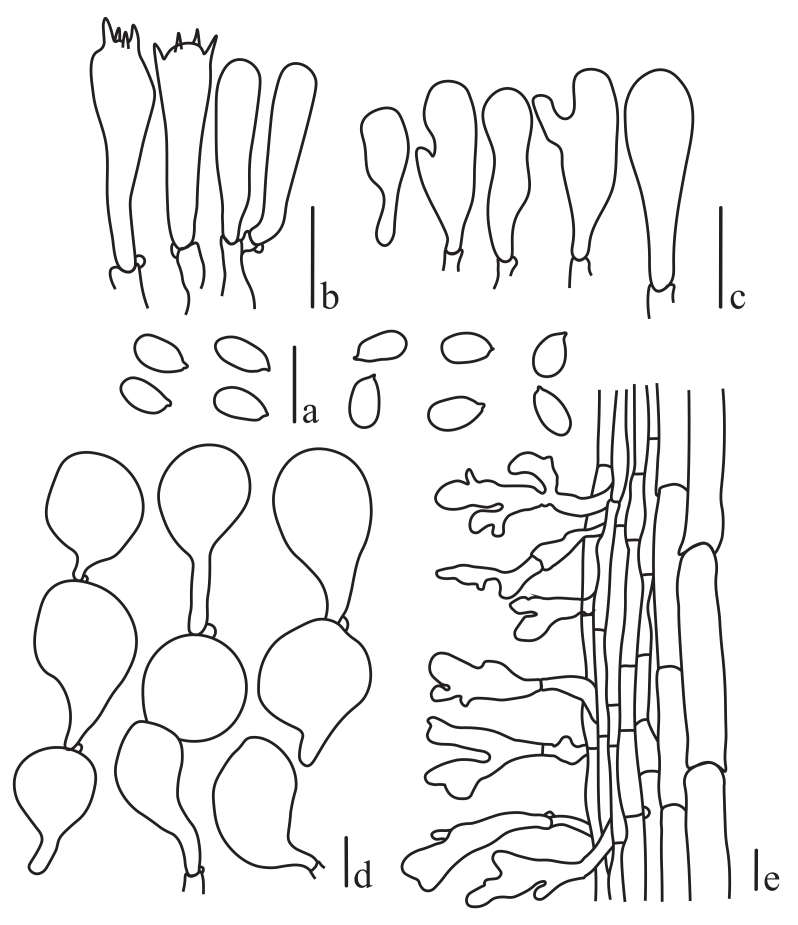
Microscopic characteristics of *Roridomyces pruinosoviscidus*. **a** Spores; **b** Basidia; **c** Cheilocystidia; **d** Pileipellis elements; **e** Stipitipellis with caulocystidia. Scale bars = 10 µm.

**Basidiomata** small. **Pileus** 2.5–11 mm diam., hemispherical to parabolic when young, expanding to flatten with age, white, with a central depression, brownish-orange (5C5); surface dull, dry, striate, pruinose; margin decurved, thin, orange-white (5A2, 6A2) to yellowish-white (2A2). **Context** thin, white. **Lamellae** decurrent, distant, with 1–3 series of rugose lamellulae; orange-white (5A2). **Stipe** 5–50 × 1–2 mm, central, equal, round and hollow; surface viscid to glutinous, shiny; apex milk-white (1A1–2), then changing to pale yellow (4A3), orange, white (5A2) to golden yellow (5B7); base hygrophanous, brownish-orange (5C4 to 6C8), with pale white tomentum. **Smell and taste**: none (Figs 4, 5).

**Bioluminescence**: the whole basidiomata and the mycelia on the PDA emit yellowish-green light and spore print (Fig. 3b).

**Basidiospores** (5.2–) 6.0–7.6 (−8.4) × 3.0–3.5 (−4.0) μm, Q = 1.36−2.47 (n = 50), Qm = 1.85, ellipsoid to elongate, hyaline, smooth, amyloid, thin-walled. **Basidia** 18–25.5 × 4.0–5.8 μm, subclavate to clavate, 4-spored, hyaline, inamyloid, thin-walled. **Cheilocystidia** 18.8–27.6 × 6.5–9.2 μm, thick-walled, irregularly clavate to furcate, occasionally subcoralloid, hyaline, inamyloid. **Pleurocystidia** absent. **Pileipellis** hymeniform, pyriform, globose, subglobose or clavate, thin-walled, hyaline and inamyloid. **Caulocystidia** 26–40.4 × 6.8–12.4 μm, thin-walled, subclavate to furcate or coralliform, inamyloid. **Clamp connections** are present in all tissues (Figs 4, 5).

#### Distribution

Asia (China and Malaysia) and Australasia (New Caledonia and Papua New Guinea).

#### Ecology

Gregarious to caespitose on decaying wood.

#### Notes

Chew et al. (2015) transferred *M. pruinosoviscida* to the *Roridomyces* genus, based on morphological and molecular data (LSU) from specimens collected in Peninsular Malaysia. Desjardin et al. (2008) found *R. pruinosoviscidus* (as *M. pruinosoviscidus*) predominantly in Australasia and South and Southern Asia. Morphological features of our specimen that correspond to *R. pruinosoviscidus* include a parabolic to convex or pulvinate, white pileus; a viscid to glutinous stipe; clavate to bifid cheilocystidia; and a hymeniform pileipellis (Chew et al. 2015). In the phylogenetic analysis of the multigene (Fig. 6a), however, only LSU sequences from the previous species, *R. pruinosoviscidus* (ACL273 and ACL 300), are available; additional collections and resequencing of type specimens are needed and should be appropriately documented.

## Analysis

Phylogeny was based on multi-locus (ITS+LSU+SSU+*tef*1-α+*rpb*2) sequence data. The dataset consisted of 135 specimens of 92 representative taxa in Mycenaceae and *Tricholoma sinoacerbum* T.H. Li, Hosen & Ting Li, *T. terreum* (Schaeff.) P. Kumm. were selected as the outgroup taxa. The aligned dataset comprised 4,772 characters, including gaps after trimming: ITS (positions 1–630 bp), LSU (631–1,456 bp), SSU (1,457–2,634 bp), *tef*1-α (2,635–3,759 bp) and *rpb*2 (3,760–4,796 bp). The phylograms of the ML and BI analyses were similar in topology. Therefore, the phylogenetic tree obtained from ML analysis was selected and presented in this study (Fig. 6). The best RAxML tree was obtained with a final ML optimisation likelihood value of -39131.034090. The matrix had 2007 distinct alignment patterns. *Mycena* clade: Our *Mycena* specimens were nested in a supported lineage (BS 100%/0.91 PP) with *M. semivestipes* (LE-BIN 3362 and HMJAU43830) and this *M. semivestipes* lineage clusters with *M. tintinnabulum* (Paulet) Quél. (H6008524 and NSK 1017255) with 88% BS and 1 PP statistical support. In addition, after comparing the morphological details of those species, coupled with BLASTn results, our *Mycena* specimens were identified as *M. semivestipes* (Fig. 6b).

*Roridomyces* clade: Our specimens (GMB1054, GMB1056 and GMBCC1068) are nested within the genus *Roridomyces*, grouped within *R. pruinosoviscidus* specimens (ACL300 and ACL273) with bootstrap support values (BS 93%/PP 0.97) and clustered with *R. mauritianus*, *R. hyllostachydis* and *R. viridiluminus* with bootstrap support values (BS 80%/PP 0.91) and, combined with morphological features, we identified it as a new record of *R. pruinosoviscidus* (Fig. 6a).

## Discussion

The current study reports on two bioluminescent mushrooms collected during an investigation of bioluminescent fungi in Yunnan Province. Based on current findings, combined with data from previously collected specimens and a comprehensive review of Lu et al. (2024b), most bioluminescent mushrooms are found to grow in tropical and subtropical climates with high humidity. These environments provide ideal conditions for the growth and reproduction of bioluminescent fungi, where moisture plays a critical role in supporting fungal metabolism and spore dispersal. The occurrence and survival of bioluminescent fungi are primarily restricted by climatic conditions, particularly temperature and humidity, which serve as the primary drivers of their ecological distribution.

Numerous studies have investigated the biological function of fungal bioluminescence, yielding several intriguing hypotheses. One commonly accepted hypothesis is that fungal bioluminescence serves as a mechanism for attracting insects and dispersing spores in low-wind conditions. This adaptation is particularly beneficial in low-wind conditions, when spreading spores would usually be challenging (Dutta et al. 2020, Ke et al. 2023, Lu et al. 2024b). Additionally, due to its link to toxicity, it has been proposed that bioluminescence might serve as a warning signal to ward off prospective fungivores. Furthermore, bioluminescence may facilitate fungal responses to oxidative stress, adding another layer of complexity to its potential functions (Dybas 2019, Dutta et al. 2020, Syed and Anderson 2021, Ke et al. 2023).

However, not all studies support the idea that fungal bioluminescence developed largely to attract insects. Some bioluminescent fungi only exhibit bioluminescence during the mycelial stage, whereas others produce it during the basidiomata developing stage (caps, gills and stipes) or both the mycelium and basidiomata stages (Nimalrathna et al. 2022, Ke et al. 2023, Lu et al. 2024b, Perry et al. 2025). In the case of *Omphalotus nidiformis*, which is well known for its bioluminescence, non-phototactic insects have been observed on its bioluminescent basidiomata. This has led some researchers to suggest that bioluminescence might be an accidental by-product of metabolism rather than a trait that evolved specifically for spore dispersal. These findings challenge the assumption that fungal bioluminescence always has an ecological purpose related to attracting insects (Weinstein et al. 2016, Nimalrathna et al. 2022, Ke et al. 2023).

Originally, bioluminescence may have evolved to dispose of waste products from metabolism, especially when reactive oxygen species were being produced. Building on this innate metabolic feature, other ecological functions (such as attracting insects for spore spread) could have evolved into an evolutionary benefit over time (Smithsonian 2024). The intricacy of evolutionary biology is underscored by this multi-layered explanation of fungal bioluminescence, which also raises the possibility that its genesis is more complex than previously believed. To deepen our understanding of the biological significance of bioluminescence, it is necessary to further research its effects on symbiotic microbiota and explore how environmental factors affect the bioluminescence characteristics of bioluminescent mushroom cultures. This knowledge will provide valuable insights into the ecological roles and adaptive functions of fungal bioluminescence, challenging us to delve deeper into this fascinating field of study.

This study has revealed the bioluminescence properties, morphological characteristics and phylogenetic relationships of two Mycenaceae species in Yunnan, China. These findings provide new insights into the diversity of bioluminescent fungi in Yunnan and their ecological roles, offering a valuable reference for the conservation of fungal diversity and bioluminescence research in the region. The environmental factors have revealed a strong connection between bioluminescence and habitat conditions, laying the groundwork for further investigation into the ecological functions and significance of bioluminescence. There is a specific correlation between bioluminescence activity and environmental factors, such as humidity and temperature. However, a comprehensive understanding of the diversity of bioluminescent fungi in Yunnan still requires further research with additional samples from various locations. This highlights the importance of ongoing research and the potential for groundbreaking discoveries in the field of bioluminescent fungi.

## Supplementary Material

XML Treatment for Mycena semivestipes

XML Treatment for Roridomyces pruinosoviscidus

## Figures and Tables

**Figure 6a.**
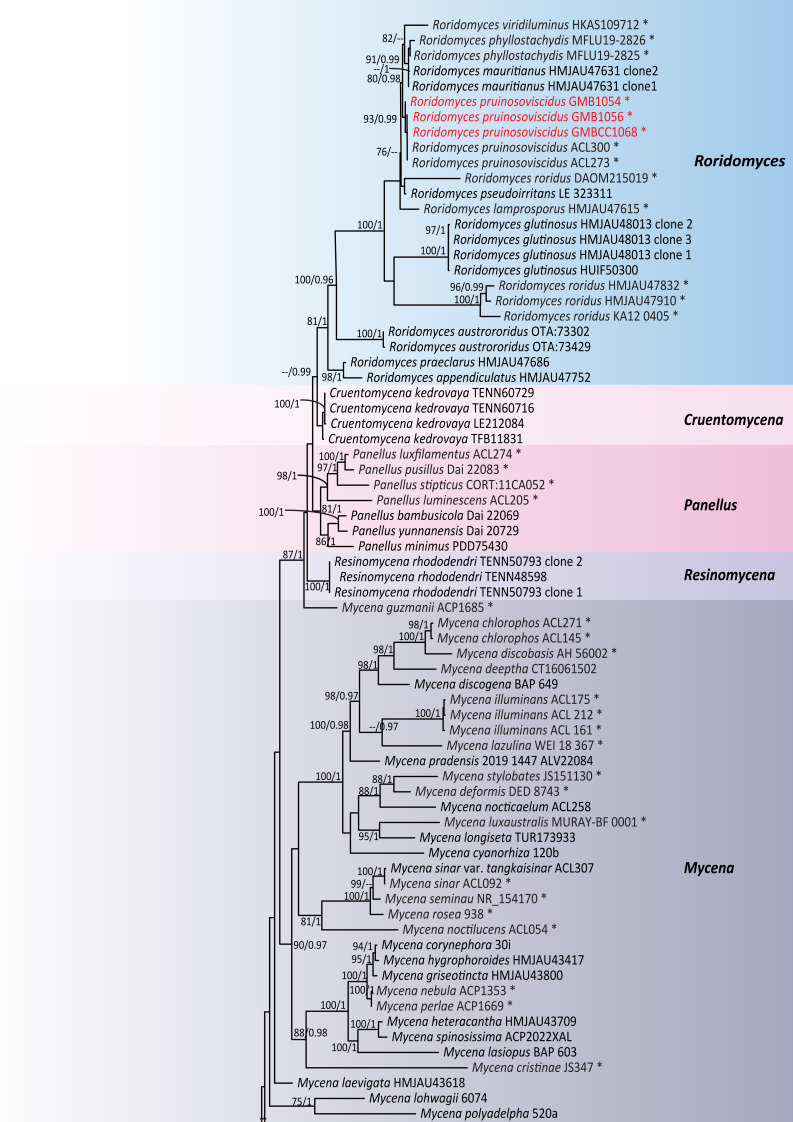
*Roridomyces* clade;

**Figure 6b.**
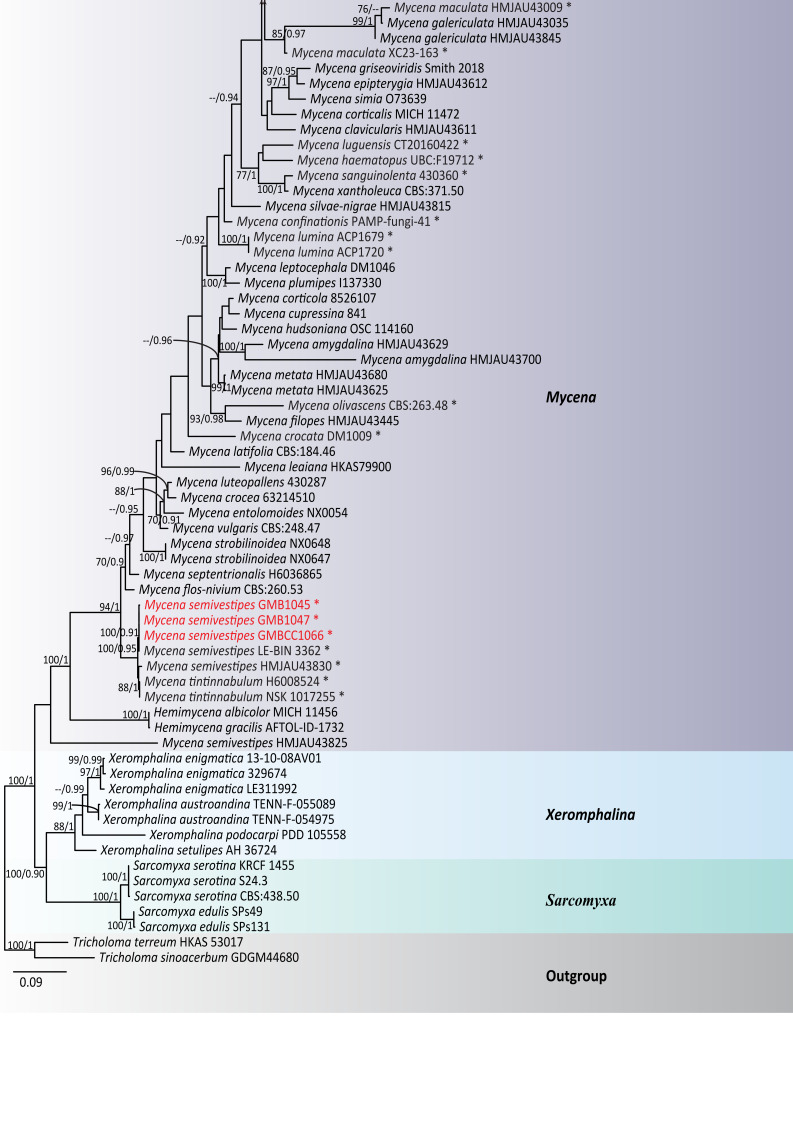
*Mycena* clade.
